# Exit point in the strong field ionization process

**DOI:** 10.1038/srep39919

**Published:** 2017-01-06

**Authors:** I. A. Ivanov, Chang Hee Nam, Kyung Taec Kim

**Affiliations:** 1 Center for Relativistic Laser Science, Institute for Basic Science, Gwangju 500-712, Republic of Korea; 2 Department of Physics and Photon Science, GIST, Gwangju 500-712, Republic of Korea; 3 Research School of Physics and Engineering, The Australian National University, Canberra ACT 2600, Australia

## Abstract

We analyze the process of strong field ionization using the Bohmian approach. This allows retention of the concept of electron trajectories. We consider the tunnelling regime of ionization. We show that, in this regime, the coordinate distribution for the ionized electron has peaks near the points in space that can be interpreted as exit points. The interval of time during which ionization occurs is marked by a quick broadening of the coordinate distribution. The concept of the exit point in the tunneling regime, which has long been assumed for the description of strong field ionization, is justified by our analysis.

An atom exposed to a strong laser field can be ionized. The Keldysh theory^1^ (also known as the strong field approximation or SFA theory) provides a basis for understanding this process and introduces the well-known classification of ionization phenomena based on the value of the Keldysh parameter 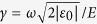. (Here *ω, E* and |*ε*_0_| are the frequency, field strength and ionization potential of the target system, expressed in atomic units). The ionization regime corresponding to the values *γ* ≫ 1 is known as the multi-photon regime. The opposite limit, *γ* ≲ 1, is known as the tunnelling regime^2^. Depending on the ionization regimes, the ionization process is described in drastically different ways^1^,^2^.

The tunnelling regime is particularly interesting since many features of tunnelling ionization and its accompanying phenomena are directly related to applications such as high harmonic generation (HHG), attosecond pulse generation and above-threshold ionization. Simple models based on the concept of electron trajectory have been developed in order to describe these phenomena. A well-known example demonstrating the great utility of such models is the famous simple man model (SMM)^2^,^3^,^4^,^5^,^6^, reproducing many qualitative features of strong field phenomena. The semiclassical TIPIS (tunnel ionization in parabolic coordinates with induced dipole and Stark shift) model^5^,^7^,^8^ is known to produce quite accurate quantitative results^5^,^6^,^7^,^9^,^10^.

In the TIPIS and similar approaches, the quantum-mechanical Keldysh theory and its modifications^1^,^11^,^12^,^13^,^14^,^15^ provide initial velocity distributions^6^,^7^,^9^ for the subsequent classical electron motion. The initial value of the coordinate is defined either by the Field Direction Model (FDM)^10^ or, in a more refined approach based on use of the parabolic coordinate system^16^, as a point at which electron emerges from under the barrier. This separation of the tunnelling ionization process in the quantum-mechanical part, describing the ionization event proper, and the classical part, describing subsequent motion, has been extremely fruitful, as it allows us to consider processes occurring in the strong laser field for systems that are too complex to allow an *ab initio* quantum mechanical (QM) treatment. It has been demonstrated^17^,^18^ that for small values of the Keldysh parameter, deep in the tunnelling regime, the results obtained using TIPIS agree very well quantitatively with the results of the Perelomov-Popov-Terentiev (PPT) theory^13^, which considers all stages of the electron motion fully quantum-mechanically. Despite this success, the concept of the electron exit point remains somewhat elusive. This is mainly due to the wave nature of QM.

In the present work we explore the view of the ionization process offered by the so-called Bohmian QM^19^. Bohmian QM introduces a well-defined notion of the electron trajectory. One need not be misled by the name into believing that Bohmian QM is something drastically different from orthodox QM. The difference between Bohmian and orthodox QM is, largely, only in the interpretation of the role of the wave-function. Bohmian mechanics reproduces exactly all the predictions of orthodox QM^20^. One does not need to subscribe to the Bohmian interpretation, moreover, to use its useful features, such as the concept of the electron trajectory. This feature has been exploited to describe ionization of atoms^21^,^22^ and molecules^23^,^24^ driven by strong laser fields, and for the description of the HHG process^25^,^26^. An approach to the problem of the tunnelling time, based on Bohmian QM, has been described recently in ref. 27. In the present work, we show that, by following the Bohmian trajectories, we can introduce the coordinate distributions describing ionized electrons. In particular, we can find a justification for the notion of the exit point.

## Theory

We recapitulate briefly a few facts constituting the basis of the Bohmian approach to quantum mechanics^19^. Substituting the polar form of the wave function of a system (we consider for simplicity a one-electron system), Ψ(***r**, t*) = *R*(***r**, t*) exp {*iS*(***r**, t*)} with *R*(***r**, t*) = |Ψ(***r**, t*)| and *S*(***r**, t*) = arg(Ψ(***r**, t*)), into the time-dependent Schrödinger equation and taking real and imaginary parts, one obtains:


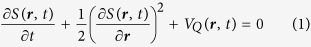



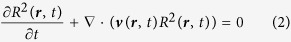


where the quantum potential and the velocity field are respectively defined as:


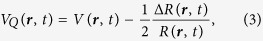


and


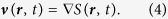


The Bohmian interpretation involves assuming that the velocity field (4) generates a family of electron trajectories for an ensemble of particles. At the initial time, *t* = 0, the coordinates of the particles constituting the ensemble are distributed as prescribed by the usual *R*^2^(***r***, 0) rule of QM. Initial velocities of the particles of the ensemble are given by Eq. (4), evaluated at *t* = 0. Electron trajectories for *t* > 0 can be found by integrating Eq. (4) along each trajectory, provided that the velocity field, ***v***(***r**, t*), is known as a function of coordinates and time. Alternatively, one may note that Eq. (1) is a Hamilton-Jacobi equation for a system described by the quantum potential (3). One may, therefore, find Bohmian trajectories by solving Newton’s equations of motion that are equivalent to the Hamilton-Jacobi equation (1), with the initial conditions specified above.

We consider a hydrogen atom in the field of a laser pulse *E*_*z*_ = *E*_0_*f*(*t*)cos *ωt*, polarized along the *z*-direction, which we use as a quantization axis. The pulse envelope function is *f*(*t*) = sin^2^ (*πt*/*T*_1_), where *T*_1_ is the total pulse duration. We performed calculations for pulses with *T*_1_ = 3*T* and *T*_1_ = 4*T*, where *T* = 2*π*/*ω* is an optical cycle (o.c.) of the field. We present results for various field strengths and frequencies, corresponding to the tunnelling regime of ionization. The initial state of the system is the ground state of the hydrogen atom. To solve the fully three-dimensional time-dependent Schrödinger equation (TDSE), we employed the procedure described in the works^28^,^29^. The atom-laser field interaction is described using the length gauge.

Using the time-dependent wave-function Ψ(***r**, t*) provided by the TDSE, we can rewrite Eq. (4) in an equivalent way as:


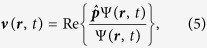


where 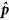 is momentum operator. This equation gives us the velocity field as a function of spatial coordinates and time. Since the wave-function in our approach is defined on a spatial grid, we obtain the velocity field at the grid-points. The velocity field at other points is found by means of the Lagrange interpolation procedure. Due to the symmetry of the problem with respect to rotations around the *z*-axis, it is sufficient to compute the velocity field in any plane containing the *z*-axis. We choose the (*x, z*)- plane for this purpose. For the initial ground state of the hydrogen atom, all the Bohmian trajectories launched at *t* = 0 have zero velocities. It is a well-known feature of Bohmian QM^19^ that the velocity field in a state described by a real wave-function is zero. The physical possibility of this state of motion in the Bohmian picture is due to the fact that the force corresponding to the quantum potential (3) vanishes for such states, allowing particles to stay at rest.

Having obtained the velocity field ***v***(***r**, t*) in the (*x, z*)- plane, we launch an ensemble (≈5 × 10^5^ trajectories) of electron trajectories. The evolution of the trajectories in time is found by numerically integrating the system of differential equations 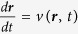 with the initial conditions *x*(0) = *x*_0_, *z*(0) = *z*_0_ in the (*x, z*)- plane.

**Figure 1:**
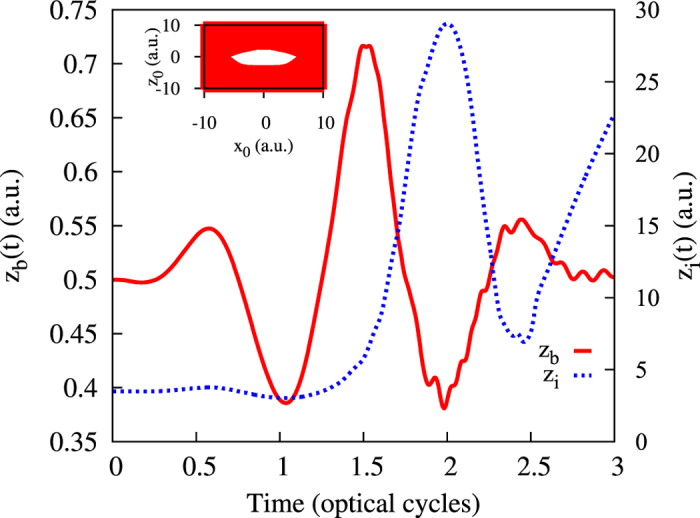
Electron coordinate along the polarization direction, as a function of time for a laser pulse with peak strength *E*_0_ = 0.0534 a.u., frequency *ω* = 0.057 a.u. and total duration of 3 optical cycles. (Red) solid line: bound trajectory *z*_*b*_(*t*) launched with initial conditions *x*_0_ = 0, *z*_0_ = 0.5 a.u. (Blue) dots: ‘ionized trajectories’ *z*_*i*_(*t*) with *x*_0_ = 0.3 a.u., *z*_0_ = 3.5 a.u. For better visibility, two vertical axes are used – the left vertical axis shows the range of the *z*-values for the bound trajectory *z*_*b*_(*t*), while the right vertical axis shows the range of *z*-values for the ionized trajectory *z*_*i*_(*t*). Inset shows dependence of the character of the electron trajectory on the initial coordinates in the (*x, z*)-plane. The initial values (*x*_0_, *z*_0_) corresponding to the ionized trajectories are in the (red) filled area.

Some of the trajectories obtained in this way describe electrons remaining bound, while some describe ionized electrons. Two typical examples for different pairs of initial conditions, *x*_0_, *z*_0_, producing bound and ionized trajectories, are shown in Fig. 1.

The overall character of the trajectories can be inferred from the inset in Fig. 1, where we left uncolored the region in the (*x, z*)-plane from which bound trajectories originate. We define ‘ionized trajectories’ here as those trajectories for which the distance of the electron from the atomic core at the end of the pulse exceeds a threshold value *R*_min_. We found that the particular value of *R*_min_ is not important, as long as the value of this parameter exceeds atomic dimensions. We use below *R*_min_ = 10 a.u.

As in ordinary statistical mechanics, an ensemble of particles can be described using distribution functions. At any time *t*_1_ > 0, a distribution function *ρ*(Ω, *t*_1_) giving the probability of detecting an electron with coordinates ***r*** and velocity ***v***, lying inside a region, Ω, of the electron’s phase-space can be found as:


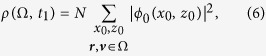


where *ϕ*_0_(***r***) is the initial ground state wave-function of the hydrogen atom, *N* is an overall normalization factor, and only trajectories ending in Ω at *t* = *t*_1_ are included in the sum. We impose one further restriction on the trajectories included into the sum (6). We are interested in those members of our ensemble for which ionization has occurred. This means that we must separate the distribution function describing the ionized subsystem from the total ensemble. This separation is necessary if the ionization probability is small and the contribution of the ionized electrons is difficult to see. To separate the ionized trajectories, we use the same criteria we employed above, including in the sum in Eq. (6) only those trajectories for which the distance of the electron from the atomic core at the end of the pulse exceeds the value *R*_min_. We should note that, with this choice of the parameter *R*_min_, the electrons which end up in the Rydberg atomic states after the end of the pulse are counted as ionized. This procedure agrees with the physical picture we are describing in the manuscript. Our aim is to follow the development of the ionization process in time. We must, therefore, take into account all the electron trajectories for which ionization event occurred at least once during the time interval of the pulse duration. It has been suggested^30^ that the dominant mechanism, leading to the population of the Rydberg states in the tunnelling regime, is the frustrated tunnelling ionization (FTI), a two-step process including tunnelling and subsequent rescattering. The majority of the electrons ending up in the Rydberg atomic states must, therefore, undergo ionization during the interval of the pulse duration.

For practical computation of the sum in Eq. (6), we launch the trajectories at time *t* = 0 with the initial conditions in phase-space region Ω_0_ = *D*_0_ × {0}, i.e. initial coordinates (*x*_0_, *z*_0_) in a region *D*_0_ of the (*x, z*)-plane, and zero velocities. For the region *D*_0_, we take a rectangle in the *x, z*-plane: |*z*| < 6 a.u., 0 < *x* < 6 a.u. The rectangle is divided into a number of squares, each with a side length of 0.01 a.u. Similarly, the phase-space volume at the time *t* = *T*_1_ at the end of the pulse is divided into a set of regions Ω_*i*_ (with each Ω_*i*_ being a direct product of squares with a side length of 0.01 a.u. in the momentum and coordinate spaces). With phase-space thus discretized, the discretized version of the distribution (6) can be obtained (apart from an overall normalization factor) as a number of trajectories arriving at the time *t* = *T*_1_ into a given region Ω_*i*_, weighted with the appropriate scaling factor, depending on the coordinate probability distribution in the initial state. We checked that the results we obtain are stable with respect to variations of the discretization parameters.

From the point of view of the statistical mechanics, the procedure encapsulated in Eq. (6) is equivalent to solving the Liouville equation for the distribution function describing the ensemble:


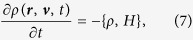


where {*A, B*} is a Poisson bracket, 
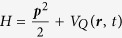
, with a quantum potential defined in Eq. (3), and a distribution function describing the ensemble at the initial time *t* = 0 given by *ρ*(***r**, **v***, 0) = |*ϕ*_0_(***r***)|^2^*δ*(***v***). We shall be interested not in the full distribution function, but in the reduced quantity *W*(*z, t*), describing the probability distribution for the electron coordinate along the laser polarization direction. This can be obtained by integrating *ρ*(Ω, *t*_1_) over all variables except *z*. A discretized version of this distribution function can be computed by including in the sum in Eq. (6) all the ionized trajectories having, at time *t*, a value of *z*-coordinate between *z* − Δ/2 and *z* + Δ/2. We use Δ = 0.01 a.u. in the calculation.

## Results and Discussion

**Figure 2:**
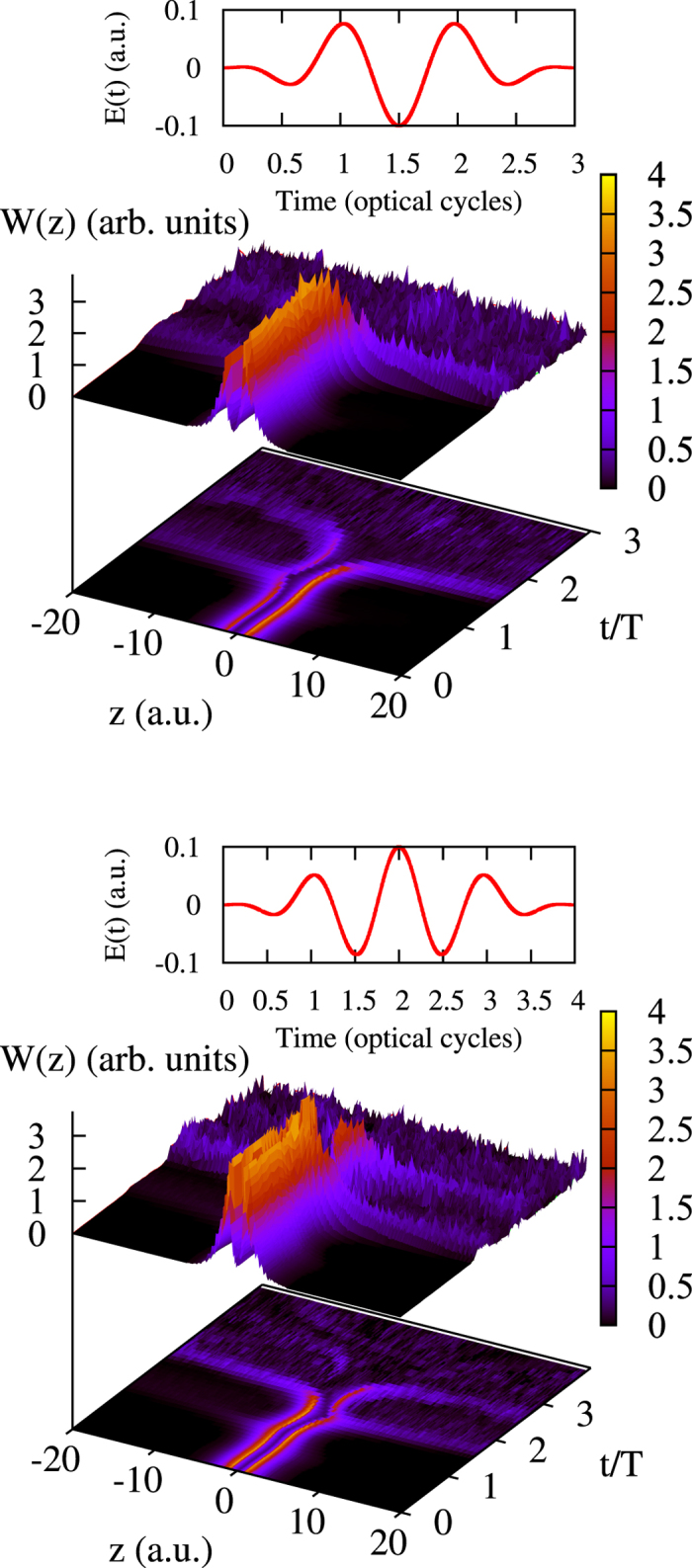
Distribution W(z) for *E*_0_ = 0.1 a.u., *ω* = 0.057 a.u. Pulse duration of 3 optical cycles (top panel), and 4 optical cycles (bottom panel). Insets show electric field of the pulse as a function of time (in units of optical cycles).

Distribution functions, *W*(*z*), calculated according to the recipe described above, are shown in Fig. 2 for the tunnelling regime of ionization (Keldysh parameter *γ* = 0.57 for the field parameters used to obtain the results presented in the Figure). One can clearly see that the distribution obtained in the tunneling regime has a double peak structure for small *t*, meaning that the ionization predominantly originates from two points along the polarization vector direction, which can be interpreted as exit points. This observation agrees with FDM.

To see how an ionized electron moves in the laser field, we follow the evolution of *W*(*z*) as time evolves. As the top panel of the Fig. 2 shows, in the case of tunneling ionization by a laser pulse with a total duration of 3 optical cycles, the distribution is a narrow-peaked function of *z* for all *t* except when the absolute value of the electric field of the pulse reaches a local maximum. In a narrow interval, including the local maximum at *t* ≈ 1.5*T, W*(*z*) undergoes a qualitative change. For this interval of time *W*(*z*) becomes a broad coordinate distribution, extending far into the region of large positive *z*-values. This long tail, which the distribution *W*(*z*) acquires, ensures that for a short period of time around the local field maximum, the probability to find an electron at the distances from the atom exceeding typical atomic dimensions increases dramatically. Natural interpretation of this behavior is to consider it as a signature of a burst of ionization in the Bohmian picture. This interpretation is further supported by the behavior of *W*(*z*) in the intervals of time containing secondary field maxima at *t* = *T* and *t* = 2*T*. In these intervals the distribution *W*(*z*) undergoes similar qualitative changes, developing tails extending far into the region of large negative *z*-values, with the implications that the probability to find an electron at large distances from the atom rises considerably. This behavior is consistent with the pulse shape shown in the inset in the top panel of the Fig. 2, electric field lowering the barrier in positive *z*-direction for *t* = 1.5*T* and negative *z*-direction for *t* = *T* and *t* = 2*T*, thus enabling electron to escape in these directions.

**Figure 3:**
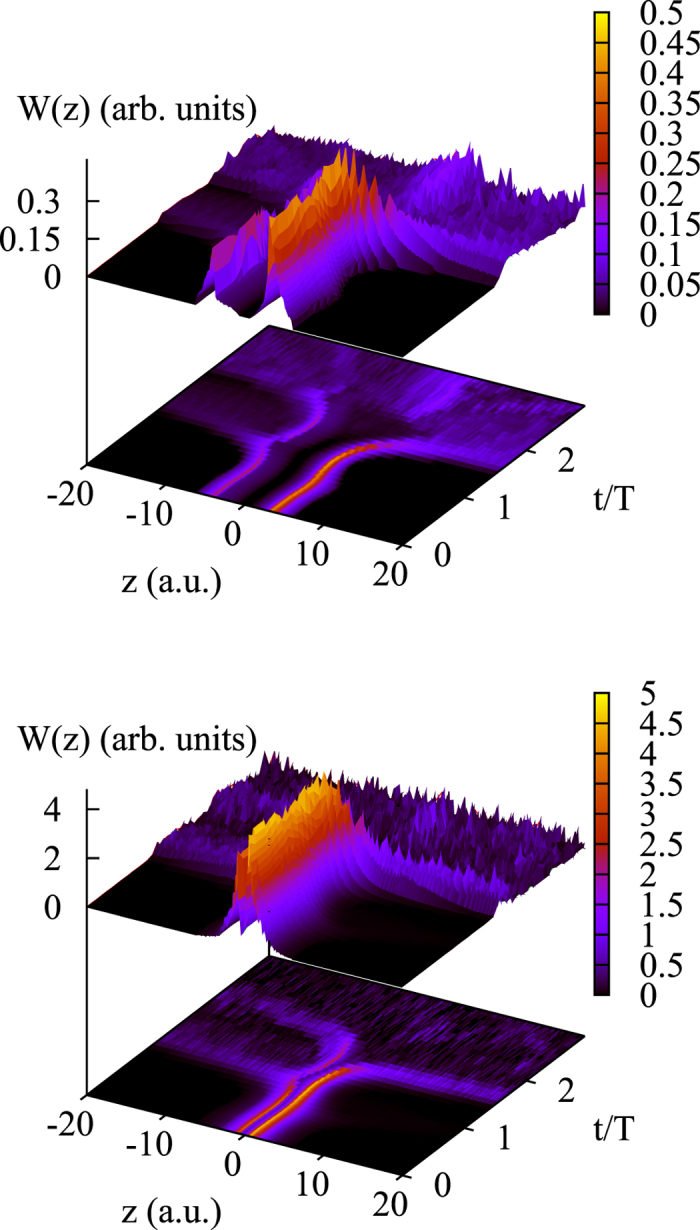
Top panel: distribution W(z) for *E*_0_ = 0.0534 a.u., *ω* = 0.057 a.u. Bottom panel: distribution W(z) for *E*_0_ = 0.12 a.u., *ω* = 0.057 a.u. Pulse duration 3 optical cycles (pulse shape shown in the inset in the top panel of Fig. 2).

We have performed analogous calculations for other field parameters (different pulse strengths and pulse durations) and found that the described behavior is quite typical for the tunnelling regime of ionization, as evidenced by the results shown in Figs 2 and 3. In all cases, the tails in *W*(*z*) appear only in relatively short intervals of time, in agreement with the well-known fact that ionization predominantly occurs in short time intervals around the peak field strength. We may interpret the appearance of these tails in the coordinate distribution as a signature of ionization bursts in the Bohmian picture of ionization. The value of *z* immediately before the time when the tails appear may then be interpreted as the exit point, i.e. the *z*-value of the electron coordinate at the time when electron exits from under the barrier. This value is to be understood in the probabilistic sense, as the distribution *W*(*z*) at the time before the electron’s exit has finite width. This width is, however, relatively small, and this justifies the concept of a well-defined exit point that is often assumed in simulations^5^,^7^.

**Figure 4:**
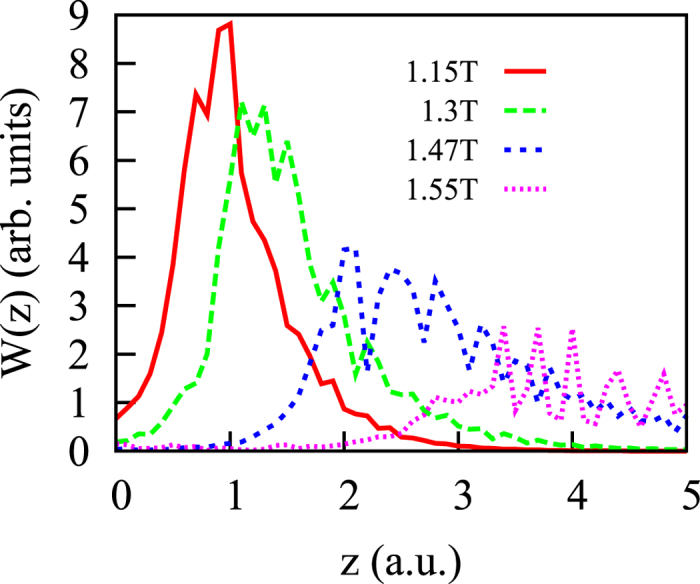
The evolution of the distribution *W*(*z*) with time. *E*_0_ = 0.1 a.u., *ω* = 0.057 a.u., pulse duration 3 optical cycles (pulse shape shown in the inset in the top panel of Fig. 2).

Figure 4 gives a more detailed view of the process of the formation of the tails in the tunnelling regime, illustrating the evolution of the distribution *W*(*z*) for different times around the mid-point of the pulse shown in the inset in Fig. 2. One can observe the fast process of the development of a tail in the coordinate distribution, *W*(*z*) undergoing a change from a narrow to a broadly-peaked function of *z* in a short interval of time around the maximum peak field strength. This agrees with the probabilistic view of the exit time^10^. As present results show, the coordinate of the exit point should also be understood in a probabilistic sense.

**Figure 5:**
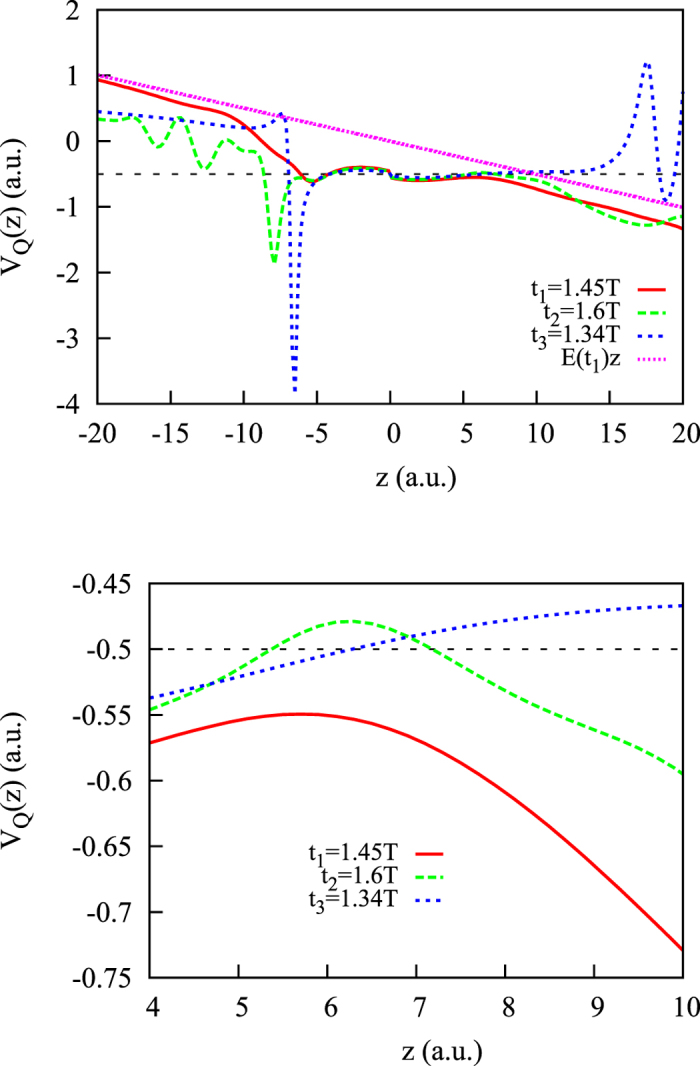
Top panel: quantum potential, *V*_*Q*_(*z, t*), evaluated along the laser polarization axis for *t*_1_ = 1.45*T, t*_2_ = 1.6*T, t*_3_ = 1.35*T* for a laser pulse of the peak strength *E*_0_ = 0.0534 a.u., where the pulse shape is shown in the inset in the top panel of Fig. 2. (Black) dashed line corresponds to the energy of the initial state. (Magenta) dotted line shows the potential *E*(*t*_1_)*z*, with electric field amplitude evaluated at the time *t* = *t*_1_. Bottom panel: potential curves under magnification.

To understand better the Bohmian perspective of the development of the ionization process in the tunnelling regime, let us consider in more detail the quantum potential given by the Eq. (3). The Bohmian trajectories are real for all the interval of the pulse duration. The tunnelling in the Bohmian picture occurs not because the trajectory at some point becomes complex (as in the quantum orbits^31^,^32^ approach), but because the quantum potential (3) effectively removes the barrier. For electric fields that are not too strong, when the depletion of the ground state can be neglected, the time-dependent wave-function of the system can be written as Ψ(***r**, t*) = *ϕ*_0_*e*^−*iεt*^ + *ϕ*_*i*_(***r**, t*), where *ϕ*_0_*e*^−*iεt*^ is the time-evolved ground state wave-function and *ϕ*_*i*_(*t*) describes the ionized wave-packet. The SFA ionization amplitudes, which are the Fourier transforms 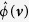 of the *ϕ*_*i*_(*t*), are essentially Gaussian functions of the velocity components^14^. The main dependence of the amplitude on the velocity in the polarization direction (which interests us presently) is given, for a hydrogen atom, by the factor^14^: 
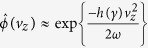
, where *h*(*γ*) = arcsinh*γ* − *γ*(1 + *γ*^2^)^−1/2^, *γ* is the instantaneous value of the Keldysh parameter. The characteristic length on which 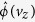 changes appreciably in *v*-space is, therefore, *a* ≈ (2*ω*)^1/2^*h*(*γ*)^−1/2^. The characteristic length on which *ϕ*_*i*_(*z, t*) changes in the coordinate space (assuming Gaussian character of 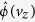) is then 2/*a*. From this estimate and from the Eq. (3), defining the quantum potential, we may deduce that as long as we stay close to the atomic core, so that |*ϕ*_*i*_(***r**, t*)| ≪ |*ϕ*_0_(***r***)|, the contribution to the quantum potential due to the ionized wave-packet is confined to the region *z* ≲ 2*a*^−1^. This point is illustrated in Fig. 5, where the snapshots of the quantum potential computed according to Eq. (3), are given for several times around the maximum field strength for the pulse with shape shown in the inset in the top panel of Fig. 2. The quantum potential *V*_*Q*_(***r**, t*) = *V*_*Q*_(0, 0, *z, t*), evaluated along the laser polarization axis, is shown.

One can see that, at time *t*_1_ = 1.45*T* near the pulse midpoint, the quantum corrections effectively remove the barrier in the positive *z*–direction, so that a classical escape trajectory is possible. (This point is better illustrated in the bottom panel of Fig. 5, which shows potential curves under magnification). For times *t*_2_ = 1.6*T, t*_3_ = 1.35*T*, farther from the pulse midpoint, the barrier is closed. The quantum corrections to the potential are due to the part of the wave-function describing the ionized wave-packet, and using the estimate we made above, we find that these corrections manifest themselves close to the atomic core in the region *z* ≲ 3 a.u. for the field parameters in the Fig. 5. These corrections may become important again near the nodes of the wave-function, where the condition |*ϕ*_*i*_(***r**, t*)| ≪ |*ϕ*_0_(***r***)| is not satisfied. Figure 5 supports these assertions. For *z*-values outside the range of the quantum corrections due to the ionized wave-packet, *V*_*Q*_(*z, t*) is (up to an insignificant constant factor) just the potential *E*(*t*_1_)*z* describing the electron interaction with the instantaneous electric field of the pulse, provided the trajectory does not pass through a node of the wave-function. As Fig. 5 shows, this is the case for the trajectory escaping at *t*_1_ = 1.45*T* in the positive *z*-direction. The electron motion along this trajectory after the ionization event can be described considering only the laser field, which is a basic assumption made in the SMM.

The coordinate of the exit point can be estimated using a variant of the FDM formula obtained using parabolic coordinates to take full account of the Coulomb atomic potential: 
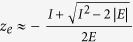
10. Here *I* is the ionization potential and *E*- is the *z*– component of the electric field at the peak strength. This formula gives *z*_*e*_ ≈ 3.6 a.u. for the field parameters in Fig. 4, in good agreement with the location of the peak of the distribution *W*(*z*) at the instant when it broadens and describes (in the picture we developed above) the time of ionization.

To summarize, we have performed an analysis of the strong field ionization process based on the Bohmian approach. An advantage offered by the Bohmian approach, which we have exploited in the present work, is the possibility of using the well-defined notion of an electron trajectory that is valid on the whole interval of the duration of the laser-atom interaction, including the interval of the sub-barrier motion. After the ionization event, the Bohmian trajectories describing ionized electrons are essentially the classical trajectories describing electron motion in a laser field. This provides a connection between the Bohmian approach and the SMM. Using the Bohmian approach, we defined the notion of the distribution of the electron coordinate in the direction of the laser field, and this sets initial conditions for the subsequent classical motion. This distribution undergoes rapid changes at times when ionization occurs, remaining a sharply-peaked function of *z* for times immediately prior to the ionization event. This can be interpreted as a justification of the notion of the electron coordinate at the exit point.

A question arises: can the results presented above be obtained using prescriptions of the conventional QM? To answer this question, one should note that the statement that the Bohmian approach leads to the same predictions as conventional QM requires a qualification. This statement is literally true, i.e. there is a one-to-one correspondence between the predictions of the Bohmian and conventional QM only when the latter provides an unambiguous answer^33^. Consider, as an example, the tunnelling time problem, which has been discussed recently in ref. 27 or transmission time, discussed in ref. 33. One can propose several definitions of the tunnelling time (e.g., Larmor time, Büttiker-Landauer time, Eisenbud-Wigner time), based on various aspects of the description of the motion based on conventional QM^27^. Analogously, several plausible definitions for the transition time can be given within the framework of conventional QM^33^. The Bohmian approach, on the other hand, leads to definitions of these concepts that are based on the the so-called ‘dwell time’ (i.e. the time a particle spends inside a given region), which in the Bohmian picture is defined in a quite natural and essentially unique way. (For discussions of the relations between Bohmian time and tunnelling and transmission times, see refs 27 and 33, respectively.) This essential uniqueness is an attractive feature of the Bohmian approach. The situation with the exit point is similar, the Bohmian approach offers the possibility of defining this notion in a natural way.

## Additional Information

**How to cite this article**: Ivanov, I. A. *et al*. Exit point in the strong field ionization process. *Sci. Rep.* **7**, 39919; doi: 10.1038/srep39919 (2017).

**Publisher's note:** Springer Nature remains neutral with regard to jurisdictional claims in published maps and institutional affiliations.
